# Granulated Wheat Biscuit

**Published:** 1875-07

**Authors:** 


### GRANULATED WHEAT BISCUIT.

An ingenious gentleman, residing in the interior of the State of New York, has conceived the idea that a much more palatable and nutritious article of food can be fabricated from wheat, than has heretofore been made. He has carried his ideas into effect, with quite remarkable results.

The chief features of his improved system are the means employed in the reduction of the grain to powder, and in the mixing and baking processes. In these items the change is radical.

He does not grind his wheat in the ordinary way, but pounds it with hammers, run by steam power. The grain runs through a " stamp-mill," similar to that used for crushing ores in mining countries.

To obviate the tendency in the ordinary wheat, to heat and "gum up," it is first dried in an oven, " kiln-dried," as it were. This prepares it for crushing, when a blow breaks the kernel and reduces it to a dry powder at once. If it is desired to make a fine and very white biscuit, the powdered wheat is sifted or " bolted," in the usual way; if, on the contrary, a darker biscuit, containing all the valuable contents of the cereal, is preferred, the powder is not subjected to any sifting process. In either event, the subsequent processes are the same.

The powder, or flour, is placed in huge troughs in which great arms ply back and forth, actuated by steam power. Water, pure and cool, is added, a little at a time, and the arms are set in motion.

By degrees, the mass becomes a stiff dough. When thoroughly mixed this dough is placed under heavy hammers, where it receives a tremendous pounding. The effect of this is to make the dough whiter in color and lighter in consistence. From a heavy, agglutinated mass, it becomes, after an hours hard beating, a light, porous and sponge-like substance. From the beating trough it passes between ponderous polished rollers, directly to the cutting-machine. The rollers having reduced it to a very thin sheet, the cutters divide the sheet into round disks or wafers, which are conveyed by an endless chain into a soap-stone oven, of peculiar construction, where they are baked.

In the whole process, the materials are not touched by human hands. This is a feature which cannot be to highly commended. The work of the ordinary baker is one of great heat, and is of course attended with profuse perspiration . It is horrible to reflect that the most hateful secretions of the human body, the exudations from those true sewers of the system—the perspiratory ducts—saturated with the waste and poison of the body—it is horrible to reflect that this poisonous matter, which would destroy life if not thrown off, is mingled with nine-tenths of all the bread wheat! The disease thus induced will never be computed, because the vastness of the evil can scarcely be comprehended. That a bread, or wheat product, at last exists which is free from this fearful contamination of the sweat of human bodies, is a consolation indeed.

We need not, perhaps enlarge upon the merits of this new food. We have been supplied with a quantity of it and have tested both the white or sifted, and the light or unsifted kinds. We find both excellent, vastly superior, in fact, to anything of the biscuit or cracker description which we have ever examined. In fact, we have, after a careful examination of the processes and the product, assured the inventor that we consider the "Granulated Wheat Biscuit," *the best food in the market.* Dyspeptics will do well to eat this food in preference to bread made by the old plan, as it will be found more palatable, more nourishing and vastly more wholesome.

This new article is being introduced to the market by "The Health Food Company," a hygienic Association, organized in New York, for the purpose of supplying wholesome cooked foods to the world. The office of this company is in the same building with our publication office and this contiguity enables us to speak with confidence and commendation of its operations.

